# Analysis of Convolutional Neural Network Segmentation Algorithm of Adenomyoma

**DOI:** 10.1155/2022/1629443

**Published:** 2022-09-14

**Authors:** Yi Jin, Wendi Huang, Qinghong Qu

**Affiliations:** The First People's Hospital of Wenling(Taizhou University Affiliated Hospital, Taizhou University Wenling Clinical Medical College), Wenling 317500, China

## Abstract

Adenomoma is a common disease occurring in the female uterus. The symptoms and pain of adenomoma seriously troubled the physical and mental health of contemporary women. However, because of the significant advantages of nondestructive and low price, ultrasound examination is used as the main imaging method for clinical diagnosis of gynecological diseases at the present stage and is often used in the initial screening and postoperative diagnosis and treatment of uterine diseases. Imaging provides a very rich information in the medical diagnosis of tumor and is a very important basis for the disease diagnosis and treatment at this stage. Ultrasound images are different from medical images such as X-ray and MRI. Because of the characteristics of imaging principles and noise interference, ultrasound images need to rely on rich clinical experience of doctors in the process of disease diseases, which increases the difficulty and work burden of doctors to some extent. Therefore, the project aims to study the deep learning segmentation method suitable for ultrasonic images. Combined with the Deeplab network in the convolutional neural network, comparing the results of the FCN network, and then finding that the Deeplab network has obvious advantages as an image segmentation model of uterine adenomyoma. In clinical practice, it can reduce the work burden of doctors and try in the direction of uterine adenomyomas ultrasound image segmentation, to fill the gap in this field.

## 1. Introduction

Adenomyoma is a benign disease in which growth-functioning endometrial glands and stromal tissue invade the myometrium, accompanied by compensatory hypertrophy and hyperplasia of the surrounding myometrium cells [1]. Adenomyomas occur more frequently in middle-aged women, and its incidence is increasing year by year [2]. In the initial screening stage of the disease, doctors will first use medical imaging examination, among which ultrasound (ultrasound, US) imaging is one of the most commonly used cross-sectional diagnostic imaging methods in medical practice, and it is also the most important auxiliary method in the diagnosis of gynecological diseases [3]. However, the ultrasound image results themselves are prone to some phenomena, such as unclear edge information, serious noise interference, and artifacts, which have high requirements on the clinical experience and discrimination ability of doctors [4]. With the application of deep learning in visual image, language processing, intelligent devices, and other fields have made breakthrough achievements. Its research in the medical field is also becoming more and more extensive, and scientists have proposed many methods of using deep models to process medical images [5]. It can not only improve the efficiency of medical image processing but also improve the processing accuracy through algorithm and preprocessing operations. Among them, the image automation and segmentation technology based on deep learning have an important research value in identifying the tumor boundary, judging the lesion volume, and tracking the postoperative follow-up.

## 2. Image Segmentation under a Convolutional Neural Network

### 2.1. Convolutional Neural Network

Convolutional neural network (CNN) is a class of feedforward neural network with convolutional operations and deep structure [6–8]. Compared with the early BP neural networks, the convolutional neural networks were succeeded in two aspects: the first is “local perception” and the second is “parameter sharing.” On the one hand, it reduces the weight for convenient optimization, and on the other hand, it simplifies the structure to prevent overfitting. In addition, CNN is also widely recognized in the field of audio recognition and natural language processing. These applications boil down to how the CNN can automatically learn features from large-scale data and generalize the results to unknown data of the same type. Then the convolutional neural network began to shine and become one of the research priorities of many scientific fields. However, the most essential feature of a neural network is that it is composed of a large number of neurons (nodes) connections, and each neuron represents a unique function, called an activation function. Two nodes are marked with weights between them, and the memory of the neural network is obtained by weighting different feature graphs [9]. The output structure of the network is affected by its internal links.

#### 2.1.1. Core Ideas

The three parts of local perception, weight sharing, and downsampling are the core ideas of convolutional neural networks [10–13].

*Local perception*. Simply put, we change the neurons' capture of the image features from the whole to the local change. In order to simplify the model to remove the redundancy and reduce the connection, we only need to let the neurons capture the local information and then summarize the captured information to obtain the global features and information.

*Ram sharing*. Weight sharing refers to the same convolution core parameter to convolute an input image, and in the process of operation, it will not lead to change the value of this parameter according to the position. The main purpose is divided into the following parts: first, when the neural network inputs a pixel value, it will correspond to a weight. After the weight is unified, the corresponding cost can reduce the cost. Second, the pixel value as input does not use the local correlation of space [11]. However, the weight sharing is precisely the use of the spatial local correlation, which is also the CNN to do the automatic feature extraction, which is a very key link. However, each convolution kernel corresponds to only one feature, and in order to strengthen the learning ability of the model, multiple cores need to be used to complete the extraction of the image feature information [12].

*Subsampling*. In the CNN, the pooling layer is the downsampling. The downsampling is to stabilize some fuzzy features to stabilize the performance of the model [13].

#### 2.1.2. Basic Structure

The convolutional neural network consists of convolutional, pooling, and fully connected layers. Because each neuron of the output feature graph in the convolutional layer will connect the input image part, forming a sparse link structure, in the training process, can avoid the fitting phenomenon and reduce the training pressure [14].

**Figure 1:**
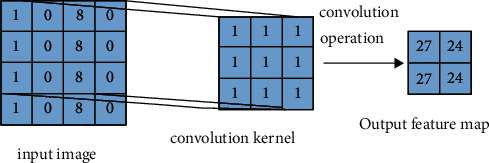
Convolution layer operation process.

*Convolution layer*. The main role is to capture features and extract features. After multiple convolution kernel operations, pictures are sent into the activation function, to realize the output of several feature maps, in the image segmentation, you can complete the capture of feature information [15].The convolution layer operation process is shown in Figure 1:

Assuming that the *i* feature graph in the convolutional layer of layer *l* is *P*_*i*_^*l*^, then *P*_*i*_^*l*^ and the m-feature graph of layer in the previous layer are *P*_*m*_^*l*−1^, and the convolution kernel between the two is *w*_*im*_^*l*^, which can be the matrix of *N* × *N*. The calculation formula is as follows:

(1) $$\begin{array}{c} P_{i}^{l}=\sum_{m=1}^{M}w_{im}^{l}.P_{m}^{l-1}+a^{l}. \end{array}$$

In the above formula, *M* represents the number of feature maps in the previous layer and *a*^*l*^ represents the fixed offset value of the *l*th convolution layer. The convolution kernel is a weight parameter matrix, and the weight sharing mentioned above is its most important feature. It can not only reduce the excess overhead of parameters, simplify the model network, optimize the training time, reduce the number and complexity of parameters, and reduce the storage overhead of the neural network.

**Figure 2:**
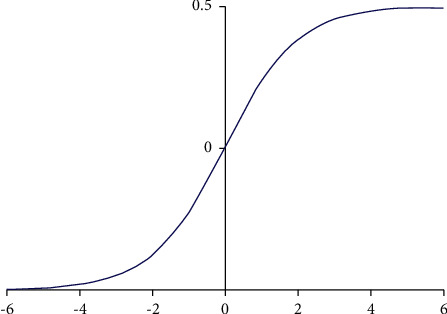
The sigmoid function image.

*Activation function* [16]. Before the formal input to the pooling layer, the output of the convolutional layer also needs to accept the following operations to change the linearity to meet the training needs of different networks and break the limitations of the linearity. Therefore, it is first transmitted to the activation functions, such as the Sigmoid function, the Tanh function, and the Re LU function. The Sigmoid function corresponds to the formula as follows, and the image is shown in Figure 2:

(2) $$\begin{array}{c} \sigma_{s}\left(x\right)=\frac{1}{1+e^{-x}}. \end{array}$$

**Figure 3:**
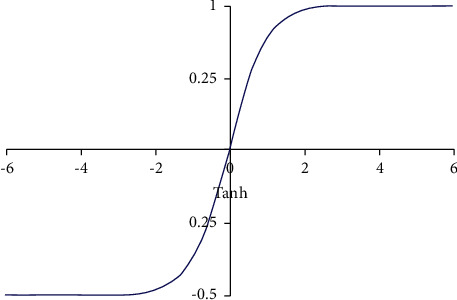
The tanh function image.

The Tanh function is as follows, and the image is shown in Figure 3:

(3) $$\begin{array}{c} \sigma_{\tau }\left(x\right)=\frac{e^{x}-e^{-x}}{e^{x}+e^{-x}}. \end{array}$$

According to the images of Figures 2 and 3, the Sigmoid function and the Tanh function can be guided everywhere, but when the input is small or large, the gradient is 0. Therefore, in the back-propagation algorithm calculation, it is easy to appear the gradient disappearance when multiplying the function bias derivative layer by layer and cannot converge the model. At this point, the gradient-vanishing problem can be solved using the Re LU function. The Re LU function refers to the linear modified cell function, and the corresponding expression is

(4) $$\begin{array}{c} f\left(x\right)=\max\left(0,x\right). \end{array}$$

**Figure 4:**
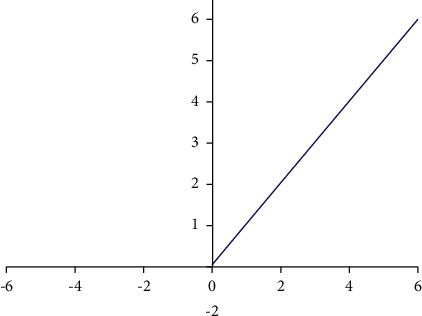
The Re LU function image.

The curve image of the Re LU function is shown in Figure 4:

We can think of the above Re LU function image as a two-part composition, with the formula: *x* represents the negative time derivative, equal to 0, representing the gradient equal to 0, in which process, the Re LU neuron turns off, and in nonnegative values, the derivative = 1. This approach can reduce the gradient-vanishing phenomenon. It also means that the neurons are sparsely activated. The Re LU functions calculate better for linear functions; for nonlinear functions, they compensate for the disappearance of the Sigmoid function gradient. Therefore, Re LU functions are often selected as an activation function [17–20].

*Pooling layer*. Pooling layer is an important part of CNN. Its main role is to reduce data, reduce parameters, and avoid risk overfitting. The pooling layer is divided into the following parts: the first role is to sample and reduce the dimension; the second role is to increase the receptive field; the third role is to ensure translation, scale and rotation invariance, and retain image features; and the fourth role is to reduce network parameters and reduce feature redundancy. Pooling is mainly divided into average pooling and maximum pooling. Max-pooling is to select the largest parameter value within the corresponding constituency, and average pooling is to select the average value within the corresponding constituency.

*Full connection layer*. After the above operation, the image features are transmitted into several fully connection layers. Each node in the fully connected layer is one-to-one to its previous layer node. The purpose of this layer is to integrate the feature parameters extracted from the previous layer, which is also the data features extracted from the data to facilitate learning.

### 2.2. Semantic Segmentation of Ultrasound Images

Semantic segmentation refers to dividing the images according to categories into areas representing different semantics and finally obtaining a segmented image with pixel-by-pixel semantic annotation. Semantic segmentation obtains refined segmentation results through pixel-by-pixel discrimination, so as to achieve the purpose of understanding the image from the pixel level. The CNN network has the characteristic of invariance, which means that, however, the semantic information of the image shifts, and the final result will not change [21].

In order to minimize the loss of location information, it is necessary to expand the size of the feature map; however, it will reduce the receptive field, so, Deeplab Empty algorithm is proposed to expand the feature graph and receptive field to obtain more context information. Deeplab is a new expanded convolution semantic segmentation model, developed by the Google team based on a convolutional neural network. To retain more edge details, Deeplab finally uses a fully connected CRF (normal conditional random field) to optimize the performance. Although slightly more computational, complex decoding structures are avoided, this study compares the results of Deeplab model with the FCN model [22].The FCN model was used for the image segmentation. Extending from the classification of whole images to the pixel-level classification is also recognized as a milestone in the field of image semantic segmentation. FCN network uses convolutional layer instead of full connection layer, making the network has greater improvement and breakthrough [23]. This way the image can vary in size at input, reducing the limitation. When it deconvolutes the feature map of the convolutional layer, the original size of the input picture can be recovered. Moreover, the prediction is generated for each pixel point, fully retaining the spatial information of the initial input image, while also reducing the overhead of the storage space. The features include the following: the convolutional layers replace the fully connected layer, upsampled, and jump connected. Full convolutional neural network is a great breakthrough in deep learning in the field of image segmentation, which realizes image pixel-level segmentation. FCN is the first fully convolutional network to be proposed and has become a classic model of semantic segmentation, but its model network also has obvious shortcomings [24]: segmentation results are relatively rough, lost details, slightly less refined, and considers less of the association between pixels. There is no fusion of the underlying information, the context of information connection is less, and the spatial consistency needs to be improved.

#### 2.2.1. Model Architecture

*Hollow convolution*. Hollow convolution introduces the concept of “expansion rate” into the traditional convolutional layer, and this parameter defines the spacing of the pixels when the convolution kernel is convoluted. While ignoring some pixels, 0 is inserted between the convolution kernel weight parameters to change the receptive field of the network using the above method [25].

*Full connection condition random airport (Dense CRF)*. Deep convolutional network (DCNN) usually has an inevitable drawback, that is, the deeper the network model layer classification, the better the effect; however, as the number of layers increase, feel the increase of the field, the result will become more smooth, location information will become blurred, and cannot clearly describe the boundary of the object [26].So, using Dense CRF, this considers the global information, refining the image edge details, and coupling them to deep convolutional neural networks for better results.

#### 2.2.2. Environmental Configuration for Ultrasound Image Segmentation of Uterine Adenomyoma

The network is trained with a single-block GPU. The specific experimental hardware configuration and software environment are shown in Table 1.

The experimental parameters were set and are shown in Table 2.

### 2.3. Construction of the Data Sets

The experiment used adenomyoma ultrasound image data set that is the hospital admitted adenomyoma patients 211 cases, each patient has 5 ∼10 ultrasound images, each ultrasound image contains transverse and longitudinal two ultrasound images, image by years of ultrasound diagnosis and adenomyosis clinical treatment experience of obstetrics and gynecology clinicians through ultrasound equipment in different patients, and has been diagnosed by the medical community gold standard, so the data set is real and reliable.

**Figure 5:**
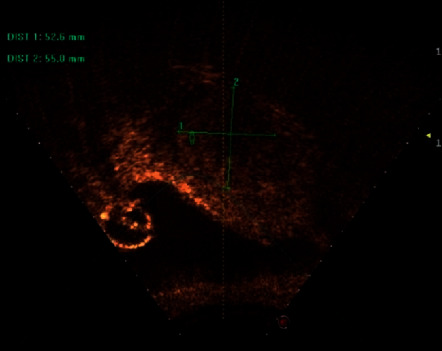
Cross of imaging of cross-imaging area.

**Figure 6:**
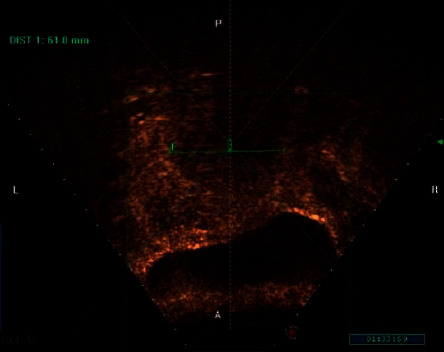
Imaginal area of longitudinal section lesions.

We selected the images of patients who were confirmed and had participated in ablation surgery and found the corresponding lesion transverse and longitudinal ultrasound images before and after surgery for sorting and matching, so as to lay a foundation for the subsequent evaluation work. The images were selected by using the GE Voluson E10 special gynecological ultrasound instrument from 211 patients with uterine adenomyomas. After high-intensity focused ultrasound treatment, the transverse and longitudinal resection image of the lesion area was complete, the relevant information of the preoperative and postoperative images was complete, and the lesion was clear and suitable for annotation. Finally, 1,589 images were selected to form the data set. Next, the image is preprocessed to remove the equipment information in the image and desensitize the patient information. Writing a series of procedures, we automatically locate the region of interest of the ultrasound image, and then the imaging area of the lesion is removed. In image batch naming for each patient's data number, distinguish the transverse and longitudinal section of tumor and distinguish preoperative images. These distinction only reflected in the naming, will not distinguish in the training model, on the one hand, to avoid useless information interference to model training, on the other hand, to facilitate the subsequent review of segmentation results. At the same time, the patient number is also a desensitization operation, to protect the privacy of patients. The processed image is as follows (Figures 5 and 6):

The labeling of the trimmed images is done under the advice and supervision of the obstetric ultrasonologist. The doctor can judge the area and shape of the lesion through the echo and mass gray scale change of the image according to the structure of the uterus and abdominal cavity. The annotation work is completed by the deep learning annotation tool Labelme. The annotation area must completely surround the outer edge of the fibroids and fit the outer outline as much as possible to ensure the accuracy of the fibroids area. In order to prevent the postoperative image correlation of the same patient, in patients, each patient corresponding 4 images (preoperative transverse images, preoperative longitudinal cutting, postoperative transverse cutting, and postoperative longitudinal cutting), a total of 170 groups of patients, the rest of the ultrasound images into the training set, to ensure the number of training set data, randomly select the effect of the test set, and to ensure the accuracy and accuracy of the model network.

### 2.4. Preprocessing of the Data

By applying geometric transformation or color transformation to the original image, more data are similar to the original data but does not change the image feature information, so that it is easier for the model to obtain the invariance features of the training data [27].The ultrasound image expansion of uterine adenomyomas should take into account the following two points:

- The amplified data set is consistent with the essential characteristics of the original data set, and it cannot change the overall statistical characteristics of the data set.
- The amplified data set should fully retain the important clinical diagnostic features such as the original lesion gray characteristics and texture information.

Therefore, the geometric transformation amplification data were selected in this study to maximize the network training advantage in the case of the limited training data set. Geometric transformation class is the geometric transformation of the image, including flipping, rotation, deformation, zoom, translation, and other operations, but because the translation and zoom operations may not guarantee the integrity of the lesion area, making the image lose the tumor disease characteristics, so it is not used in this data set enhancement.

In general, most images can be processed, and the image is enhanced by denoising algorithm and improving contrast. Uterine ultrasound images have disadvantages such as low contrast, severe noise pollution, and insignificant features. Routine mean filtering denoising method is an important link in general image processing, but these methods are harmful, can destroy the image texture features of uterine gland fibroids, and then affect the image semantic segmentation results, such as filtering image boundary area will further smooth, lose more features of lesion tissue information, difficult to feature extraction in the network training. In the experiment, we have tried to denoise the image and then conduct the network training, and the effect is not satisfactory, so the preprocessing method of image denoising is not adopted in this experiment.

### 2.5. Evaluation Indicators

*MIoU*. The evaluation of semantic segmentation [28–38] standard metric for MIoU, through the intersection of the two numerical sets and set ratio to represent the accuracy, in the two sets are true value and predicted value, the proportion of the two can be expressed as true, false negative, false-positive sum, then calculated in each category of IoU mean.

(5) $$\begin{array}{c} MIoU=\frac{1}{k+1}\sum_{i=0}^{k}\frac{p_{ii}}{\sum_{j=0}^{k}p_{ij}+\sum_{j=0}^{k}p_{ij}-p_{ii}}. \end{array}$$

Class 1 MIoU calculation process is as follows, on *i* = 1, *p*_ii_ represents the “real,” which belongs to class 1 and prediction is 1 class, among them, this belongs to 1 class is predicted for other class of pixels is ∑_*j*=0_^*k*^*p*_1*j*_, this belongs to other class is predicted for class 1 pixels is ∑_*j*=0_^*k*^*p*_*i*1_, in the denominator, calculated the *p*_11_ twice, so to subtract a *p*_11_.

*PA(Pixel Accuracy)*. PA is mainly used to predict the proportion of the correct number of pixels to the total pixel number. The calculation formula is as follows:

(6) $$\begin{array}{c} PA=\frac{\sum_{i=0}^{k}p_{ii}}{\sum_{i=0}^{k}\sum_{j=0}^{k}p_{ij}}. \end{array}$$

To evaluate the segmentation accuracy of the uterine adenomyomas images, we will use both PA and MIoU and simultaneously observe the actual effect of the image set to comprehensively compare the effects of the model.

## 3. Experimental Results and Model Evaluation

After data preprocessing on the image training set of adenomyomas, it is input into the Deeplab network for training, and then the test set is input into the network to get the segmentation results. The segmentation annotated data are taken as the “gold standard” to judge the quality of the model for algorithm evaluation. Although the data set is relatively small in sample, the method of transfer learning is used in the experiment, transferring the training weight logs and parameters of the large-scale data set, and applying the previous training weights in the large-scale data set to the new training. With relatively small data, good training results are achieved. Avoiding the convergence problem of the parameter model, thus improves the model segmentation accuracy.

**Figure 7:**
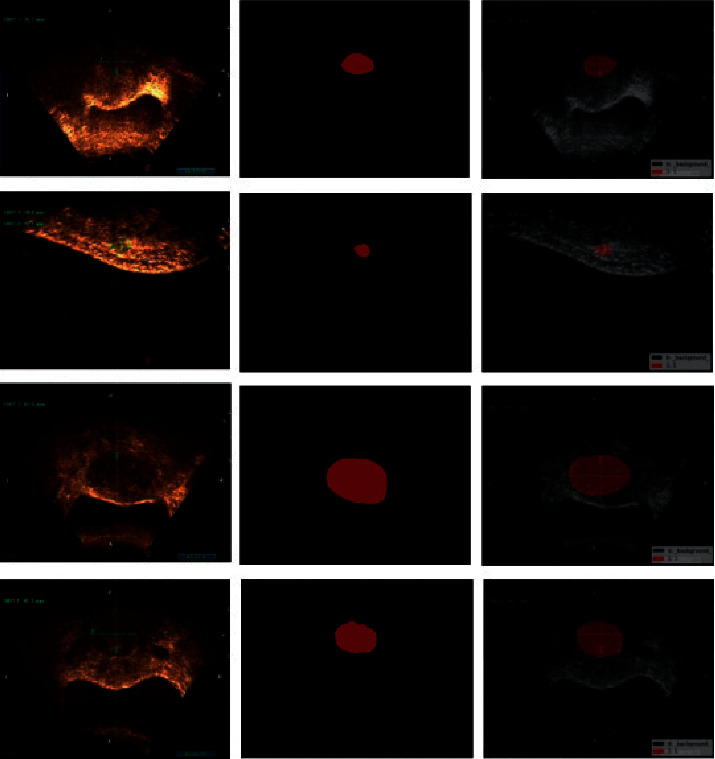
The Deeplab test set is an output result control.

In this experiment, the same experimental hardware and software conditions were used to compare other semantic segmentation networks and compare the results. The resulting model training results are shown in Table 3:

In the training of the FCN model, the deconvolution step needs to be set to 32 to directly expand 32 times when upsampling, and the effect is poor. Therefore, continue the 8 times upsampling to fuse the prediction results of the third pooling layer, the step length is set to 8, and then get the FCN model results, so as to get better results. The overall training time limit is not high. And the intersection ratio value did not significantly improve, and the overall effect is general. However, using the Deeplab network as an image segmentation model for uterine adenomyoma has obvious advantages.  Figure 7 shows the actual test set using Deeplab network:

Ultrasonic images of the original tumor mask plot of the lesion segmentation image of lesion annotation.

In the experiment, the tumor pixels were set to class 1, and the background map was set to class 0. Because the number of tumor pixels was much larger than the background pixels and the data balance difference between classes, the final segmentation accuracy of the tumor pixels was 0.7277, and the overall pixel accuracy of the image reached 0.9893. Thus, the Deeplab semantic segmentation network segments the ultrasound images of adenomyomas.

Lesis segmentation plays an essential role in the diagnosis and treatment of adenomas. The Deeplab segmentation network proposed in this paper realizes the automatic segmentation of ultrasound images of adenomas and improves the efficiency of initial diagnosis and treatment. According to the data characteristics and the actual diagnosis and treatment needs, the data image was preprocessed to form a data set for uterine adenomyoma lesion segmentation. The network parameters were then set to train the model and finally the segmentation results of the FCN model on the uterine adenomyoma image test set were compared. The experimental results show that Deeplab network has great segmentation advantages, with its advantages of small cost, high accuracy, and high boundary fine degree to prove that this model is more suitable for ultrasonic image segmentation of uterine adenomyomas, with an average intersection ratio of 84.76%, which can achieve more accurate segmentation effect. And compared with other models, it has the advantages of low cost, high accuracy, and easy to implement.

## 4. Conclusion

Because of the disadvantages of ultrasonic image noise and artifacts, its development in the field of artificial intelligence image segmentation. Different from superficial diseases in vivo, ultrasound tumor images in the abdominal cavity with unclear boundaries and large interference also make it difficult for segmentation. In order to solve the above problems, reduce the work burden of doctors and meet the needs of rapid and efficient clinical diagnosis, and this study tries in the direction of ultrasonic image segmentation of adenomyomas to fill the gap in the application of deep learning algorithm in this field. After data preprocessing on the image training set of adenomyomas, it is input into the Deeplab network for training, and then the test set is input into the network to obtain the segmentation results. The segmentation annotated data are taken as the “gold standard” to judge the quality of the model for algorithm evaluation. Although the data set sample is relatively small, the transfer learning method is used to transfer the training weight logs and parameters of the large-scale data set network and to use the previous training weights of the large-scale data sets in the new training session. With a relatively small amount of data, very good training results were obtained. Avoid reinitializing the parameter model that fails to converge successfully and improve the segmentation accuracy of the model. According to the experimental demonstration, the algorithm proposed in this paper is applicable to the clinical auxiliary diagnosis system of uterine adenomyomas, and it also has certain value in the clinical application perspective, which is helpful for doctors to quickly identify the focal area without being limited by clinical experience.

## Figures and Tables

**Table 1:** Experimental software and hardware Configuration.

GPU	AMD ryazen 7 1800X
GPU	NVIDIA GeForce GTX 1060
Internal storage	16G
Deep learning framework	TensorFlow 1.12.0
Develop the interface	Python 3.6
Operating system (OS)	Windows10

**Table 2:** Experimental parameters.

Batch size	4
Batch number	36675
Maximum iteration times	50
Weight decay	0.0005
Learning rate	0.00025

**Table 3:** Results of Deeplab model and other semantic segmentation models (U-Net).

Evaluating indicator	Deeplab	FCN
MIoU	84.76%	56.47%
PA	0.9893	0.8533

## Data Availability

The data set can be obtained from the corresponding author upon request.
